# Development of a real-time multiplex PCR assay for the detection of multiple *Salmonella *serotypes in chicken samples

**DOI:** 10.1186/1471-2180-8-156

**Published:** 2008-09-21

**Authors:** Edel O'Regan, Evonne McCabe, Catherine Burgess, Sheila McGuinness, Thomas Barry, Geraldine Duffy, Paul Whyte, Séamus Fanning

**Affiliations:** 1 Centres for Food Safety and Food-borne Zoonomics, UCD Veterinary Sciences Centre, University College Dublin, Belfield, Dublin 4, Ireland; 2 Ashtown Food Research Centre, Teagasc, Ashtown, Dublin 15, Ireland; 3 Department of Microbiology, National University of Ireland Galway, Galway, Ireland

## Abstract

**Background:**

A real-time multiplex PCR assay was developed for the detection of multiple *Salmonella *serotypes in chicken samples. Poultry-associated serotypes detected in the assay include Enteritidis, Gallinarum, Typhimurium, Kentucky and Dublin. The traditional cultural method according to EN ISO 6579:2002 for the detection of *Salmonella *in food was performed in parallel. The real-time PCR based method comprised a pre-enrichment step in Buffered Peptone Water (BPW) overnight, followed by a shortened selective enrichment in Rappaport Vasilliadis Soya Broth (RVS) for 6 hours and subsequent DNA extraction.

**Results:**

The real-time multiplex PCR assay and traditional cultural method showed 100% inclusivity and 100% exclusivity on all strains tested. The real-time multiplex PCR assay was as sensitive as the traditional cultural method in detecting *Salmonella *in artificially contaminated chicken samples and correctly identified the serotype. Artificially contaminated chicken samples resulted in a detection limit of between 1 and 10 CFU per 25 g sample for both methods. A total of sixty-three naturally contaminated chicken samples were investigated by both methods and relative accuracy, relative sensitivity and relative specificity of the real-time PCR method were determined to be 89, 94 and 87%, respectively. Thirty cultures blind tested were correctly identified by the real-time multiplex PCR method.

**Conclusion:**

Real-time PCR methodology can contribute to meet the need for rapid identification and detection methods in food testing laboratories.

## Background

Contaminated poultry products are widely accepted as a major source of *Salmonella *infections [1]. The annual cost of medical treatment for salmonellosis, in addition to loss of productivity, imposes a significant financial burden on many countries. At present more than 2,500 serotypes of *Salmonella *are known. Serotypes Enteritidis and Typhimurium accounted for the majority of cases of human salmonellosis in Ireland in 2006 [2]. A report on Zoonoses in Ireland in 2004 shows that of the 7,616 raw poultry meats sampled at processing level, 245 (3.2%) were positive for *Salmonella *with the most common serotypes isolated being Enteritidis, Kentucky, Bredeney and Mbandaka [3]. The results of the European baseline survey on the prevalence of *Salmonella *in broiler flocks in 2005–2006 indicated 27.9% positive flocks in Ireland, compared to 23.7% in the EU overall [4]. While the prevalence of *Salmonella *in egg-laying flocks was 1.4% in Ireland according to the European baseline study, compared to 30.7% in the EU overall [5].

Currently, international guidelines and regulations for the detection of *Salmonella *sp. in foods are based on traditional cultural methods, which takes at least 5 days for confirmation of results [6]. More recently attention has focused on molecular based methods due to their sensitivity, specificity and reduced assay time. Conventional PCR based assays for *Salmonella *detection in foods have been widely reported [7-10]. Additionally real-time PCR assays for the specific detection of *Salmonella *are increasingly documented [11-19]. Both of these methods for detection of *Salmonella *in foods have been brought to inter-laboratory trial, the results of which support their use as international standard methods [20,21]. Real-time multiplex PCR assays for simultaneous detection of two or more genera in foods e.g. *Salmonella *and *Campylobacter *in chicken rinse fluid [22] and *Salmonella *and *Listeria *in raw sausage meat [23,24] have been described. The *invA *gene target is most commonly used for the detection of *Salmonella *in PCR assays, however gene targets such as *ttrRSBCA*, *sipBC *and *stn *have been used as well [10-15,17-23]. A duplex real-time PCR assay for the detection of *Salmonella *Enteritidis in poultry meat and consumption eggs has been developed with primers and *Taq*Man probes based on the *Salmonella *specific *invA *gene and the *prot6e *gene located on the *S*. Enteritidis specific 60 kb virulence plasmid [24]. To date, conventional multiplex PCRs for serotyping in clinical isolates have been described using the *rfb *locus and flagellar alleles as gene targets, however they have not been tested on food samples [25-27].

Rapid pathogen testing is vital to the food industry and facilitates increased public health protection. Real-time PCR methodology reduces the reporting time of results compared to the traditional microbiology method. These methods can prove advantageous to food manufacturing companies by preventing costly and damaging product recalls, as most food products are not held in warehouses pending test results.

In this paper we report the development of a real-time multiplex PCR assay for the detection of multiple *Salmonella *serotypes in chicken samples and assess its equivalence with the traditional cultural method, ISO 6579 (2002). The multiplex real-time PCR assay comprises four targets, *aceK*, which is *Salmonella *sp. specific and three other targets that are *Salmonella *serotype specific; *sefA *specific for serotypes Enteritidis, Dublin and Gallinarum:*sdf *target specific for *Salmonella *Enteritidis only and *fliC *target specific for serotypes Typhimurium and Kentucky.

## Methods

### Bacterial strains used in this study

Sixty *Salmonella *strains and thirty non-*Salmonella *strains used in the selectivity testing in this study are listed in Tables 1 and 2, respectively. *Salmonella *Enteritidis ATCC 13076, *Salmonella *Typhimurium ATCC 14028, *Salmonella *Kentucky NCTC 05799 and *Salmonella *Bredeney NCTC 05731 were used to artificially contaminate chicken samples. These serotypes were chosen for artificial inoculation experiments because they have been associated with poultry-related outbreaks of gastroenteritis in Ireland [28,29].

**Table 1:** *Salmonella *strains used in the selectivity testing of real-time multiplex PCR assay and traditional cultural method. C_T _values are given in parentheses.

*Salmonella *serotypes	ISO 6579	Real-time multiplex PCR			
		*aceK*	*fliC*	*sefA*	*sdf*
Agona	+^a^	+ (23.31)	-^b^	-	-
Anatum	+	+ (21.67)	-	-	-
Braenderup	+	+ (21.24)	-	-	-
Bredeney	+	+ (21.71)	-	-	-
Derby	+	+ (22.79)	-	-	-
Dublin (n = 7)^c^	+	+ (17.63)	-	+ (19.35)	-
Enteritidis (n = 11)	+	+ (21.35)	-	+ (23.36)	+ (23.67)
Gallinarum (n = 3)	+	+ (26.87)	-	+ (27.83)	-
Goldcoast	+	+ (24.35)	-	-	-
Hadar	+	+ (19.68)	-	-	-
Heidelberg	+	+ (21.12)	-	-	-
Infantis	+	+ (23.34)	-	-	-
Kentucky (n = 8)	+	+ (18.59)	+ (21.65)	-	-
Livingstone	+	+ (21.90)	-	-	-
London	+	+ (19.92)	-	-	-
Manhattan	+	+ (17.53)	-	-	-
Newport	+	+ (18.04)	-	-	-
Nottingham	+	+ (21.89)	-	-	-
Panama	+	+ (19.54)	-	-	-
Saintpaul	+	+ (21.06)	-	-	-
Senftenberg	+	+ (21.01)	-	-	-
Stanley	+	+ (19.23)	-	-	-
Typhimurium (n = 10)	+	+ (19.77)	+ (22.45)	-	-
Uganda	+	+ (20.21)	-	-	-
Virchow	+	+ (22.52)	-	-	-
Gaminara	+	+ (21.03)	-	-	-

^a ^+ = *Salmonella *positive by the method.

^b ^– = *Salmonella *negative by the method.

^c ^= Mean C_T _values are given when n > 1.

**Table 2:** Non-*Salmonella *strains used in the selectivity testing of the real-time multiplex PCR assay and traditional cultural method.

Non-*Salmonella *Strains	Type Strain Number
*E. coli*	ATCC 25922
*E. coli*	NCTC 09001
*E. coli*	NDC 544
*C. freundi*	NCTC 09750
*C. freundi*	NCTC 8090
*C. diversus*	CCFRA 7119
*C. koseri*	NCTC 10768
*E. cloacae*	NCTC 11933
*E. cloacae*	NCTC 10005
*E. agglomerans*	NCTC 09381
*E. intermedius*	NDC 427
*E. aerogenes*	NCTC 10006
*E. sakazakii*	NCTC 11467
*E. faecium*	ATCC 35667
*E. faecalis*	NCTC 12697
*B. cereus*	NCTC 07464
*K. oxytoca*	ATCC 43086
*K. pneumoniae*	ATCC 13883
*P. aeruginosa*	NCTC 12903
*P. putida*	ATCC 49128
*L. planatarum*	ATCC 8014
*S. haemolyticus*	ATCC 29970
*S. epidermis*	Unknown
*S. saprophyticus*	ATCC 15305
*S. lactis*	NCDO 2003
*A. hydrophilia*	ATCC 35654
*A. globiformis*	ATCC 8010
*L. mesenteroides*	ATCC 8293
*A. calcoaceticus*	ATCC 23055
*P. mirabilis*	DSM 4479

### Traditional cultural method and real-time multiplex PCR assay

**Figure 1:**
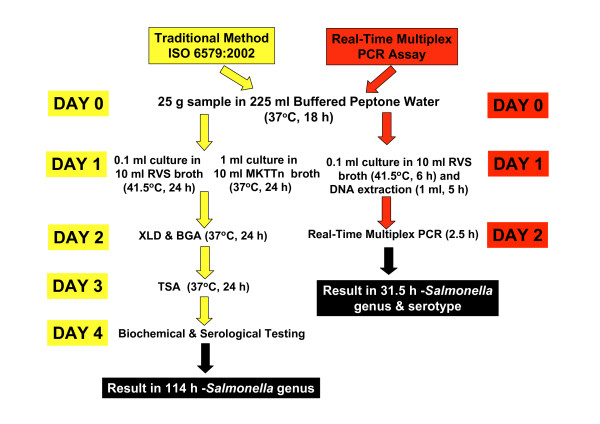
Flow diagram of methodology in the traditional cultural method and the real-time multiplex PCR assay.

All *Salmonella*, non-*Salmonella *strains and chicken samples used in this study were tested for *Salmonella *by the ISO 6579:2002 European International Standard Method [6] (Figure 1). Biochemical testing was performed using api20E test strips (bioMerieux, Marcy l'Etoile, France). Serotyping was performed according to the Kauffmann-Whyte typing scheme using slide agglutination with standard antisera (Murex, Dublin, Ireland). The real-time multiplex PCR method comprised an 18 h pre-enrichment in buffered peptone water (BPW CM1049, Oxoid, Basingstoke, UK) at 37°C, followed by 6 h incubation in Rappaport-Vassiliadis Soya (RVS) Peptone Broth (CM0866, Oxoid) at 41.5°C, with subsequent DNA extraction (Figure 1).

### Sample DNA Extraction for real-time multiplex PCR assay

DNA was extracted from pure cultures of bacteria and chicken samples that had been enriched at 37°C for 18 h in BPW and at 41.5°C for 6 h in RVS. One ml of the RVS broth was collected in an eppendorf tube, centrifuged at 15,339 × *g *for 5 min and the supernatant discarded. The cell pellets were resuspended in 1 ml phosphate buffered saline (PBS, Oxoid), centrifuged at 15,339 × *g *for 5 min and the supernatant discarded. DNA extractions were performed on the cell pellet using the DNeasy Blood and Tissue kit (Qiagen, Hilden, Germany) according to manufacturer's instructions. DNA preparations were stored at -20°C until use.

### Primers and probes used in real-time multiplex PCR assay

**Figure 2:**
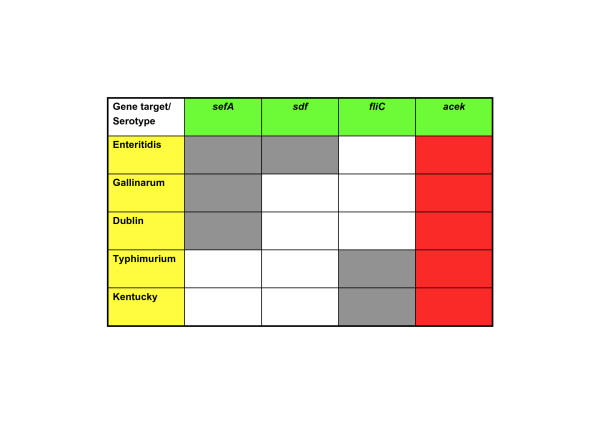
Gene targets used in real-time multiplex PCR assay and the *Salmonella *serotypes they detect.

The design of the primers was based on the multiple alignment of *Salmonella *and non-*Salmonella *sequences of the individual genes using MegAlign Lasergene software (DNAstar, Madison WI, USA). Sequences were taken from GenBank accession numbers in the literature and nucleotide entries on NCBI website . The gene targets used for primer design and for the real-time multiplex PCR assay and the *Salmonella *serotypes they detect are included in Figure 2. Four primer pairs were designed using Primer Select DNAStar Lasergene software. The specificity of the primer sequences was tested by homology searches in the nucleotide database (NCBI, nucleotide BLAST (blastn)). TIB Molbiol (Berlin, Germany) designed the *Taq*Man probes and the probes were labeled with reporter dyes FAM (*aceK*), YAK (*fliC*), ROX (*sefA*) and Cy5 (*sdf*). Primers and probes were purchased from TIB Molbiol and their sequences are presented in Table 3.

**Table 3:** Oligonucleotide primers and probes used in real-time multiplex PCR assay.

Target gene	Primer or Probe	Sequence (5'-3')	Tm (°C)	Amplicon Size (bp)	Reference or Accession No.
*sefA*	Forward	GTGGTTCAGGCAGCAGTTACT	61.1	334	L11008
	Reverse	CAGGGACATTTAGCGTTTCTTGAG	59.7		
	Probe	ROX-CAGCTCAGAATACAACATCAGCCAACTGG-BBQ	66.2		
*fliC*	Forward	CCCCGCTTACAGGTGGACTAC	60.2	433	AY649721
	Reverse	AGCGGGTTTTCGGTGGTTGT	63.6		
	Probe	YAK-TAAAGCCGCATTGACAGCAGCAGGTG-BBQ	69.8		
*aceK*	Forward	CCGCGCTGGTTGAGTGG	62.0	240	U43344
	Reverse	GCGGGGCGAATTTGTCTTTA	60.3		
	Probe	FAM-AACCACTGCCGAACTGTATATGGCGA-BBQ	65.0		
*sdf*	Forward	AAATGTGTTTTATCTGATGCAAGAGG	57.6	299	[35]
	Reverse	GTTCGTTCTTCTGGTACTTACGATGAC	58.7		
	Probe	Cy5-CGAATGGTGAGCAGACAACAGGCTGATTTA-BBQ	68.4		This study

### Real-Time Multiplex PCR assay

Real-time multiplex PCR reactions were performed with the Rotor Gene 3000 (Corbett Research, Australia) using a total volume of 25 μl, which was contained within a 0.2 ml thin-walled PCR tube. The optimal amplification reaction mixture contained 12.5 μl 2 × QuantiTect Multiplex PCR No ROX mastermix (Qiagen) containing HotStar *Taq *polymerase, QuantiTect multiplex PCR buffer, dNTP mix including dUTP and 11 mM MgCl_2_, a final concentration of 0.4 μM for each primer and probe, 4.5 μl sterile RNase-free water and 2 μl of test or control DNA. Each real-time PCR assay systematically included six control reactions performed in parallel with the test samples. These positive and negative controls used as template, genomic DNA of five *Salmonella *strains or water respectively. Incubation conditions consisted of an initial denaturation at 95°C for 15 min; followed by 35 cycles of 94°C for 60 sec and 60°C for 90 sec. Fluorescence signals were detected in FAM, YAK, ROX and Cy5 channels. The Rotor Gene 3000 analyzer is capable of detecting four dyes simultaneously in a multiplex assay. The line for calculating the threshold cycle number (C_T_) for each channel was assigned to a fixed value intersecting the amplification curves in the linear region of the logarithmic plot. Any sample showing a fluorescence signal above this line was regarded as positive. Each PCR reaction gave a positive or negative result at this threshold line. Samples with C_T _values of less than 35 cycles were considered as positive from preliminary optimization experiments.

### Selectivity testing

Sixty *Salmonella *strains (10 CFU/ml) and thirty non-*Salmonella *(1,000 CFU/ml) strains were tested by both the traditional cultural method and real-time multiplex PCR assay. The strains were cultured in 10 ml BPW at 37°C for 18 h prior to decimal dilution in Maximum Recovery Diluent (MRD CM0733, Oxoid) to obtain the required inoculation level. One ml of this dilution was then transferred to 9 ml BPW at 37°C for 18 h and a 0.1 ml aliquot of this was added to 10 ml RVS broth at 41.5°C for 6 h. One ml of the RVS broth was then collected in an eppendorf tube for DNA extraction as previously described and subsequent real-time multiplex PCR. A 2 μl aliquot of the DNA extract was used as template in the real-time multiplex PCR assay.

### Artificial contamination of chicken skin and chicken meat

Pre-packed whole chickens were purchased from local supermarkets and were tested for the presence of *Salmonella *sp. by enrichment of 25 g chicken skin and chicken meat in 225 ml BPW (Oxoid) at 37°C for 18 h, and subsequent plating on Xylose Lysine Desoxycholate agar (XLD CM0469, Oxoid) at 37°C for 18 h. As no *Salmonella *sp. was detected, the chicken was used for subsequent artificial contamination experiments.

Chicken skin and chicken meat samples (25 g portions) were artificially contaminated individually with four *Salmonella *serotypes (Enteritidis ATCC 13076, Typhimurium ATCC 14028, Kentucky NCTC 05799 and Bredeney NCTC 05731) at four levels of contamination (0, 1–10, 10–100, 100–1,000 CFU/25 g). The four strains were cultured in 10 ml BPW at 37°C for 18 h prior to decimal dilution in MRD (Oxoid) to obtain the four different levels of contamination. Twenty-five gram portions of chicken samples were inoculated with 1 ml of each level of inoculation, placed in stomacher bags and 225 ml of BPW was added. The mixture was homogenized in a stomacher (Seward, London, United Kingdom) for 1 min and immediately incubated at 37°C for 18 h. The precise numbers of CFU introduced into the chicken skin and chicken meat was determined by plating each level of inoculation on Tryptone Soya Agar plates (TSA CM0131, Oxoid), followed by incubation at 37°C for 24 h. After pre-enrichment in BPW, the cultures were subdivided into two aliquots of RVS broth: one for analysis by the traditional culture method and the second for the real-time multiplex PCR assay. A total of thirty-two chicken skin and chicken meat samples, were analyzed in triplicate by both the ISO 6579 and real-time multiplex PCR methods.

### Naturally contaminated chicken skin samples

A total of sixty-three 25 g chicken skin samples from naturally contaminated whole chickens, crowns, legs and breasts were obtained from local butchers in the Dublin area. All samples were tested for *Salmonella *by both the ISO 6579 method and real-time multiplex PCR assay.

### Blind testing of cultures

Thirty cultures on solid media were received for blind testing from a collaborating group to assess the real-time multiplex PCR assay. One ml of an overnight BPW culture of the blind samples was transferred to 9 ml BPW at 37°C for 18 h and a 0.1 ml aliquot was added to 10 ml RVS broth at 41.5°C for 6 h. One ml of the RVS broth was then collected in an eppendorf tube for DNA extraction as described previously and subsequent real-time mutiplex PCR.

### Statistical analysis and terms used

Relative sensitivity, specificity and accuracy were calculated according to the MICROVAL protocol [30]. The formulas used for the analysis were:

(1) Relative accuracy: $\text{AC}=\frac{\left(\text{PA}+\text{NA}\right)}{\text{N}}\times 100\%$

(2) Relative specificity: $\text{SP}=\frac{\text{NA}}{\text{N-}}\times 100\%$

(3) Relative sensitivity: $\text{SE}=\frac{\text{PA}}{\text{N+}}\times 100\%$

[ Where;

PA is the positive agreement between culture and PCR methods;

NA is the negative agreement between culture and PCR methods;

PD are the false positives by PCR method;

ND are the false negatives by PCR method;

N is the total number of samples (NA+PA+PD+ND);

N- is the total number of negative results with the reference method (NA+PD) and

N+ is the total number of positive results with the reference method (PA+ND)].

Relative sensitivity is the ability of the alternative method (real-time multiplex PCR) to detect the analyte when it is detected by the reference method (traditional culture method ISO 6579) in the presence of a biological matrix. Relative specificity is the ability of the real-time multiplex PCR to not detect the target organism when it is not detected by the reference method. The relative accuracy is the degree of correspondence between the response obtained by the alternative method and the reference method on identical samples [30].

## Results

### Selectivity study

The sixty *Salmonella *strains were confirmed as *Salmonella *positive by the traditional cultural method. These strains, comprising 26 different *Salmonella *serotypes, all demonstrated positive results for the *aceK *target in the real-time multiplex PCR assay (Table 1). Testing of the *fliC *serotype specific target yielded positive results for serotypes Typhimurium and Kentucky only and negative results for all other *Salmonella *serotypes tested. The *sefA *serotype specific target was positive for serotypes Enteritidis, Dublin, and Gallinarum and gave negative results for all other *Salmonella *serotypes. The *sdf *serotype specific target was specific for all *Salmonella *Enteritidis strains only and was not amplified in the other *Salmonella *serotypes tested. The thirty pure culture non-*Salmonella *strains listed in Table 2 yielded negative results by the traditional cultural method. The non-*Salmonella *strains also yielded negative results by the four gene targets *aceK*, *sefA*, *sdf *and *fliC *in the real-time multiplex PCR assay. The selectivity study verified that only *Salmonella *strains were detected by both methods and that the gene targets *sdf, sefA *and *fliC *also correctly identified the serotype where appropriate. The selectivity study also confirmed that no cross reactivity ocurred with the non-*Salmonella *strains tested.

### Detection of *Salmonella *in artificially contaminated samples

All artificially contaminated chicken samples tested positive for *Salmonella *by both the traditional cultural method and real-time multiplex PCR and all un-inoculated control samples tested negative by both methods (Table 4). The *Salmonella*-positive chicken samples were identified correctly at the serotype level by the real-time multiplex PCR assay. Mean C_T _values obtained by the detection channels ranged from 19 to 28 cycles. The C_T _values given cannot be used for quantitative interpretations because of the enrichment of each sample before the real-time multiplex PCR assay. The results from the artificially contaminated samples indicated that both methods are sensitive and able to detect to a level as low as 1–10 CFU of *Salmonella *in 25 g of chicken skin and chicken meat. The results from the multiplex real-time PCR assay were in complete agreement with the ISO 6579 method in three independent artificial inoculation experiments.

**Table 4:** Results of the real-time multiplex PCR assay compared to the traditional cultural method for the detection of *Salmonella *from artificially contaminated chicken skin and chicken meat samples

Food Type	*Salmonella *serotype	Estimated CFU by plating	ISO 6579	Real-time multiplex PCR			
				*aceK*	*fliC*	*sefA*	*sdf*
Chicken skin	Enteritidis	0	-^a^	-	-	-	-
		3	+^b^	+ (28.14)	-	+ (27.70)	+ (26.99)
		32	+	+ (24.94)	-	+ (25.15)	+ (24.51)
		168	+	+ (22.52)	-	+ (22.32)	+ (21.68)
Chicken meat	Enteritidis	0	-	-	-	-	-
		5	+	+ (20.58)	-	+ (20.09)	+ (19.88)
		36	+	+ (20.20)	-	+ (19.96)	+ (19.57)
		222	+	+ (19.67)	-	+ (19.67)	+ (19.05)
Chicken skin	Typhimurium	0	-	-	-	-	-
		3	+	+ (24.12)	+ (23.46)	-	-
		34	+	+ (22.18)	+ (21.00)	-	-
		257	+	+ (21.95)	+ (19.57)	-	-
Chicken meat	Typhimurium	0	-	-	-	-	-
		7	+	+ (23.19)	+ (22.75)	-	-
		54	+	+ (21.39)	+ (21.19)	-	-
		305	+	+ (20.84)	+ (20.42)	-	-
Chicken skin	Kentucky	0	-	-	-	-	-
		6	+	+ (26.28)	+ (25.94)	-	-
		48	+	+ (24.25)	+ (23.70)	-	-
		280	+	+ (22.12)	+ (21.41)	-	-
Chicken meat	Kentucky	0	-	-	-	-	-
		5	+	+ (23.55)	+ (22.33)	-	-
		52	+	+ (21.15)	+ (19.93)	-	-
		277	+	+ (20.57)	+ (19.49)	-	-
Chicken skin	Bredeney	0	-	-	-	-	-
		6	+	+ (22.46)	-	-	-
		60	+	+ (21.58)	-	-	-
		397	+	+ (22.00)	-	-	-
Chicken meat	Bredeney	0	-	-	-	-	-
		9	+	+ (22.37)	-	-	-
		75	+	+ (21.69)	-	-	-
		338	+	+ (22.23)	-	-	-

C_T _values, in parentheses, are means of triplicates at a fixed threshold.

^a ^– = *Salmonella *negative by the method.

^b ^+ = *Salmonella *positive by the method

### Detection of *Salmonella *in naturally contaminated samples

Fifteen of the sixty-three naturally contaminated chicken skin samples were *Salmonella *positive by both methods (PA) and all fifteen samples were identified as either serotypes Typhimurium or Kentucky by the real-time multiplex PCR (Table 5). Fourty-one of the sixty three samples yielded negative results for *Salmonella *by both methods (NA). One sample was *Salmonella*-positive by the traditional cultural method and *Salmonella *negative by the real-time multiplex PCR (ND). Six samples were positive for *Salmonella *by real-time multiplex PCR and negative by the traditional cultural method (PD). Of these six samples positive for *Salmonella *only by real-time multiplex PCR, four were identified to the serotype level as Typhimurium or Kentucky as positive signals were detected in the YAK channel as well as the FAM channel. Mean C_T _values obtained by the detection channels ranged from 19 to 32 cycles. The C_T _values given cannot be used for quantitative interpretations because of the enrichment of each sample before real-time PCR. Relative accuracy, relative sensitivity and relative specificity of the real-time PCR method were determined to be 89, 94 and 87%, respectively.

**Table 5:** Results of the traditional cultural method and real-time multiplex PCR assay for *Salmonella *detection from naturally contaminated chicken skin samples.

		ISO 6579	
		+^a^	-^b^
Real-time Multiplex	+	15	6^c^
PCR	-	1	41

^a ^+ = *Salmonella *positive by the method.

^b ^– = *Salmonella *negative by the method.

^c ^= Four of these samples were identified at the serotype level as Typhimurium or Kentucky.

### Detection of *Salmonella *in blind samples

As part of the blind testing, there was a discrepancy in results with one of the blind samples (BS11) which was identified correctly by the real-time multiplex PCR (Table 6). BS11 was identified as *Salmonella*-positive in the FAM channel of the real-time multiplex PCR assay and confirmed by biochemical and serology testing. However the colloborating group reported that it was a non-*Salmonella *isolate.

**Table 6:** Results of the real-time multiplex PCR assay for the detection of *Salmonella *from blind culture samples compared to the reported identification from another laboratory.

Lab. ID	Real-time multiplex PCR				Real-time Multiplex PCR Result	Reported Identification by other lab
	*aceK*	*fliC*	*sefA*	*sdf*		
BS1	+^a ^(23.94)	-^b^	-	-	*Salmonella*	Gaminara
BS2	+ (24.01)^c^	-	+(23.01)	-	Gallinarum/Dublin	Dublin
BS3	-	-	-	-	ND^d^	Non-*Salmonella*
BS4	+ (24.16)	-	-	-	*Salmonella*	Branderup
BS5	+ (24.77)	-	-	-	*Salmonella*	Manhattan
BS6	+ (23.89)	-	-	-	*Salmonella*	Nottingham
BS7	-	-	-	-	ND	Non-*Salmonella*
BS8	+ (25.36)	-	-	-	*Salmonella*	Anatum
BS9	+ (22.93)	-	-	-	*Salmonella*	SanDiego
BS10	+ (24.02)	+ (20.40)	-	-	Typhimurium/Kentucky	Kentucky
BS11^e^	+ (23.10)	-	-	-	*Salmonella*	Non-*Salmonella*
BS12	+ (23.78)	-	-	-	*Salmonella*	London
BS13	+ (24.67)	-	-	-	*Salmonella*	Virchow
BS14	+ (24.79)	-	-	-	*Salmonella*	Livingstone
BS15	-	-	-	-	ND	Non-*Salmonella*
BS16	+ (22.90)	-	-	-	*Salmonella*	Derby
BS17	-	-	-	-	ND	Non-*Salmonella*
BS18	+ (23.42)	-	+ (22.03)	-	Gallinarum	Gallinarum
BS19	+ (24.33)	-	-	-	*Salmonella*	Saintpaul
BS20	+ (25.45)	-	-	-	*Salmonella*	Agona
BS21	-	-	-	-	ND	Non-*Salmonella*
BS22	+ (23.04)	+ (20.55)	-	-	Typhimurium/Kentucky	Typhimurium
BS23	+ (22.59)	+ (20.03)	-	-	Typhimurium/Kentucky	Typhimurium
BS24	+ (23.59)	-	-	-	*Salmonella*	Hadar
BS25	+ (23.49)	-	-	-	*Salmonella*	Heidelberg
BS26	+ (23.12)	-	-	-	*Salmonella*	Newport
BS27	+ (23.73)	+ (22.68)	-	-	Typhimurium/Kentucky	Typhimurium
BS28	+ (23.96)	-	-	-	*Salmonella*	Reading
BS29	+ (22.56)	+ (21.35)	-	-	Typhimurium/Kentucky	Typhimurium
BS30	+ (22.79)	-	-	-	*Salmonella*	Infantis

^a ^+ = *Salmonella *positive by method.

^b ^– = *Salmonella *negative by method.

^c ^= C_T _values in parentheses.

^d ^ND = *Salmonella *not detected.

^e ^= BS11 was identified as *Salmonella*-positive by *aceK *target and confirmed by biochemical and serology testing, while the another laboratory reported that it was a non-*Salmonella *isolate.

## Discussion

The aim of this study was to develop a rapid detection method for multiple *Salmonella *serotypes based on real-time multiplex PCR and to compare it to the traditional cultural method. The detection method contained a two-step enrichment procedure comprising a non-selective and a selective enrichment step to detect *Salmonella *in chicken samples. The overnight pre-enrichment step was required to increase the number of viable cells and to effectively dilute inhibitory substances present in the sample. The use of the second selective enrichment for 6 hours also dilutes PCR inhibitors possibly derived from the food matrix and suppresses the growth of background flora. This was followed by DNA extraction and real-time multiplex PCR. Potential inhibitory substances in foods, including fats, glycogen, organic and phenolic compounds, can affect the PCR reaction [31-33]. However several techniques can be employed to overcome this problem such as simple dilution of the sample. Methods that use secondary enrichment steps will, in general, be less prone to inhibitory effects from the sample matrix than those performed from a primary culture [32].

For the development of the real-time multiplex PCR assay, primers were designed for four targets, one specific for *Salmonella *species and the other three targets were serotype specific; detecting serotypes commonly reported in Ireland. The *aceK *gene was chosen as a target for the detection of *Salmonella *sp. in the real-time multiplex PCR assay and its specificity was confirmed with at least thirty non-*Salmonella *strains in this study. The *aceK *gene encodes a bi-functional regulatory enzyme (isocitrate dehydrogenase kinase/phosphatase, IDH K/P) that catalyzes phosphorylation and dephosphorylation of isocitrate dehydrogenase and thereby controls the flux of isocitrate through the tricarboxylic acid cycle and the glyoxylate bypass [34]. The *fliC *target is specific for serotypes Typhimurium and Kentucky, which both encode the i-antigen specific phase 1 flagellin and both are poultry-associated serotypes. Further, the i-antigen is also expressed in uncommon serotypes such as Aberdeen, Bergen and Kedougou. The gene target *sdf *was chosen from the literature, it was identified by subtractive hybridization and is specific for serotype Enteritidis [35]. *sdf *is chromosomally encoded, yet its function is unknown [35]. *Salmonella *Enteritidis is most common serotype reported in poultry-associated gastroenteritis outbreaks worldwide [36]. *sefA *encodes the SEF14 fimbrial antigen and the distribution of SEF14 fimbriae is limited to a subset of group D *Salmonella *[37]. The *sefA *target detects serotype Enteritidis, hence it corroborates the results from the *sdf *target. *sefA *also detects the poultry-associated serotype Gallinarum. It detects serotype Dublin also, although this serotype is more commonly associated with cattle, it has been found on occasion in poultry samples. The *sefA *target may also identify infrequent *Salmonella *serogroup D serotypes such as Rostock, Berta, Pullorum and Seremban. This is the first report wherein these four gene targets were incorporated into a unified real-time multiplex PCR assay for the detection of multiple *Salmonella *serotypes in chicken samples.

Selectivity testing of both methods yielded 100% inclusivity and 100% exclusivity. The *Salmonella *strains used for the selectivity testing represented *Salmonella *serotypes that have been frequently reported in Ireland over the past ten years according to the annual reports on salmonellosis by the Health Protection Surveillance Centre. The non-*Salmonella *strains chosen for the selectivity testing were representative of food-borne pathogens and found in the same environments as *Salmonella*. Blind testing of cultures by the real-time multiplex PCR method indicated that the gene targets in the real-time multiplex PCR method correctly identified the thirty blind culture samples. Both methods allowed sensitive detection of *Salmonella *in artificially contaminated chicken samples, yielding positive results even at the lowest contamination levels tested (1–10 CFU/25 g chicken samples).

Data from fifty-six of the sixty-three naturally contaminated chicken skin samples were in agreement by both methods. One of the samples tested positive by traditional culture and negative by real-time multiplex PCR. This may have arisen as a result of dislodgement of the pellet during a washing step with PBS. Six of sixty-three naturally contaminated samples were positive by the real-time multiplex PCR assay and negative by the traditional cultural method. This may be attributable to the fact that target cells may have been injured or died despite the enrichment procedures i.e. were non-culturable but detectable by PCR. Similar to our findings, Schrank *et al *[38] and Loftstrom *et al *[39] noted that PCR following enrichment detected more *Salmonella *in poultry samples compared to conventional culture. Furthermore, it was reported that to be able to distinguish between salmonellae and other bacteria growing on selective solid agar, the number of *Salmonella *cells must be at least 10^4 ^CFU/ml after enrichment in tetrathionate broth (TTB) before they are detectable on selective solid media [40]. This could explain culture negativity in the samples that are PCR positive. *Salmonella *was detected in the presence of a large background flora in the naturally contaminated chicken skin samples, which was subsequently identified by biochemical testing as *E. coli*, *Enterobacter cloacae*, *Klebsiella pneumoniae*, *Citrobacter freundi *and *Pseudomonas*.

The use of an internal amplification control (IAC) is becoming mandatory for diagnostic PCR testing of food-borne pathogens as it indicates the presence of DNA polymerase inhibitors, errors caused by PCR components or malfunctions of the thermal cycler [41]. In this real-time multiplex PCR assay the simultaneous detection of *Salmonella *specific *aceK *target sequence functions as a surrogate IAC for *Salmonella *DNA in the sample. However in the event of chicken samples being negative for all four targets in the real-time PCR assay, the possibility remains that inhibitors are present in the extracted DNA leading to a false negative result that cannot be excluded.

## Conclusion

This study reports a rapid real-time multiplex PCR assay using four primer sets and four *Taq*Man probes for the detection of multiple *Salmonella *serotypes. The assay performed equally as well as the traditional cultural method and facilitated the sensitive detection of *Salmonella *in artificially contaminated chicken skin and chicken meat samples. However, further experiments should focus on inclusion of a universal IAC into the real-time multiplex PCR assay, automation of the DNA extraction, analysis of a larger number of naturally contaminated samples including additional food types and an inter-laboratory trial. The real-time multiplex PCR assay has the potential to be used in routine diagnostic laboratories when it is necessary to identify the serotype for surveillance studies and as a rapid screening tool in food testing laboratories to quickly identify *Salmonella*-positive samples.

## Authors' contributions

EO'R designed and carried out all the experimental work. EO'R drafted the manuscript. EMcC, CB and SMcG provided some of the bacterial strains used in this study and collaborated on the culture enrichment studies. SF, PW, GD and TB conceived this study. All authors read and approved the final manuscript.
